# Editorial: Towards behavior maintenance processes

**DOI:** 10.3389/fpsyg.2023.1194832

**Published:** 2023-05-02

**Authors:** Navin Kaushal, Chun-Qing Zhang, Cleoputri Yusainy

**Affiliations:** ^1^Department of Health Sciences, School of Health & Human Sciences, Indiana University, Indianapolis, IN, United States; ^2^Department of Psychology, Sun Yat-sen University, Guangzhou, China; ^3^Department of Psychology, University of Brawijaya, Malang, Indonesia

**Keywords:** physical activity, exercise, maintenance, theory, behavior change

Behavioral theories have been insightful in helping us understand how to promote health behaviors, such as increasing vaccination rates (Rosenstock, 1974) or taking steps to quit smoking (Velicer et al., 1992). Although most health-related behaviors require repeated performance, some marker of behavior maintenance is necessary to reap beneficial health outcomes, such as regular participation in physical activity, substituting alcoholic beverages to maintain sobriety, or incorporating hygiene practices. While several models have been formulated to predict behavior change (Glanz and Bishop, 2010; Davis et al., 2015; Hagger et al., 2020), very few have operationalized behavior maintenance. This Research Topic showcases seven articles that advance our understanding of how we can transition successfully adopted behaviors into long-term maintenance, which include four longitudinal studies (Hou et al.; Jiménez-Zazo et al.; Wang et al.; Zhang et al.); two reviews (Rhodes; Rhodes and Sui); and one conceptual analysis (Zhao et al.), with a focus ranging from theoretical advancement to application and testing in clinical populations.

One narrative review (Rhodes and Sui) examines concepts and theoretical approaches used to identify physical activity maintenance. The authors provide an overview of proposed definitions of maintenance, including a decrease in behavioral lapses (Spruijt-Metz et al., 2015), a time frame such as 6 months (Velicer et al., 1992; Marcus et al., 2000), and a shift in decisional focus (Rothman, 2000). The definitions are followed by an examination of the characteristics among recent behavioral models (e.g., Rothman et al., 2009; Caldwell et al., 2018; Strobach et al., 2020), where the authors highlight one commonality, namely, that behavioral maintenance is represented by a greater reliance on automatic/reflexive constructs such as habit (Gardner, 2015; Wood and Rünger, 2015; Hagger, 2019) and identity (Rhodes et al., 2016). However, a certain amount of behavioral repetition under favorable circumstances would be required to cultivate maintenance constructs, such as habit formation (Kaushal and Rhodes, 2015). Rhodes and Sui advance previous definitions of maintenance by proposing that it is reflected by a shift in mechanisms of action. The review concludes by encouraging investigation into the constructs, conditions, and timeframes associated with the achievement of maintenance. These insights, however, call attention to a set of new questions, such as whether some populations are more predisposed than others to physical activity lapses or maintenance, and what we know about physical activity maintenance in people with chronic disease.

Jiménez-Zazo et al. shed some light on the first investigation with the EXERNET study, an 8-year longitudinal study that aimed to understand physical activity adherence among older adults using the transtheoretical model of change (Prochaska and Velicer, 1997). After observing participants for eight years, the authors found that the inability to maintain physical activity was predicted by lower scores on fitness parameters such as lower body strength and aerobic endurance. These findings suggest that older adults who score low on these fitness parameters, which are related to mobility efficiency, experience greater challenges in maintaining traditional physical activity, which hinders their progress toward achieving behavior maintenance.

Two longitudinal studies examined behavior maintenance processes in patients with chronic conditions. Wang et al. integrated the Theory of Planned Behavior (Ajzen, 1991) and Temporal Self-Regulation Theory (Hall and Fong, 2007) to understand physical activity maintenance in individuals with coronary artery disease. While the authors found correlational support of habit and intention with behavior, the subsequent model test did not find habit to moderate between intention and behavior. These findings indirectly encourage structuring the model to align with the dual process frameworks (Evans, 2008) by situating habit and intention as parallel determinants of behavior, which is well-supported in the physical activity literature (Rhodes et al., 2019). While articles that promote physical activity generally focus on aerobic exercise, there is significant importance and need for resistance training in clinical populations. For instance, individuals with cancer experience skeletal muscle depletion, the effects of which can be exacerbated by chemotherapy (Gilliam and St. Clair, 2011; Aversa et al., 2017), making regular participation in strengthening exercises imperative for these patients. The work by Zhang et al. is one of the most recent articles to promote the maintenance of upper-limb exercise in post-operative breast cancer patients. The authors formulated a conceptual model that merged the Health Action Process Approach (Schwarzer, 1992) with the Theory of Planned Behavior (Ajzen, 1991) and marked intention to parse behavioral initiation and maintenance phases. Results revealed coping planning, maintenance self-efficacy, and recovery self-efficacy to demonstrate the predictive effects of physical activity maintenance.

The second half of this issue focuses on construct functionality and processes. One study (Hou et al.) employed the Health Action Process Approach model (Schwarzer, 1992) to understand the exercise intention-action (behavior) link and to determine the moderating role of self-efficacy over 12 months. The authors found that participants with high levels of self-efficacy exhibited stronger relationships between intention and planning and between planning and action phases. Self-efficacy was also found to function as a moderator between intention and planning and between planning and action stages. Another work (Zhao et al.) provided a conceptual analysis on the compensatory belief construct, which stems from motivational dissonance or confliction (Knäuper et al., 2004). A compensatory health belief is when an individual believes that indulging in a proximal unhealthy choice can be compensated for by subsequent healthy behavior(s). While there is some appeal to this notion as the individual eventually performs the planned behavior (Zhao et al.) caution that compensatory health behaviors are controversial, as many studies have demonstrated a negative relationship between compensatory behaviors and motivation.

This issue concludes with a review (Rhodes) entitled “*Multi-Process Action Control (M-PAC) in physical activity: a primer*,” which presents a viable model specific to physical activity promotion. The model design is supported by theoretical premises outlined in a recent review (Rhodes and Sui), which states that early behavioral enactment relies on regulatory processes that should be replaced by a reflexive process, which is indicative of achieving behavior maintenance. Consistent with other studies on this issue and the work mentioned in Rhodes and Sui's review, the model suggests that in addition to phase-exclusive constructs, some constructs are necessary to remain activated across both phases. Rhodes encourages future research to explore the dynamic relationship between reflexive and regulatory constructs and to implement the M-PAC for physical activity interventions.

The articles in this issue present valuable tools, updated theoretical findings, and applications to help advance our understanding of behavior maintenance. Although this issue attracted submissions that focused on physical activity maintenance, we encourage researchers to investigate behavior maintenance for minimizing unhealthy food choices, reducing sedentary behavior time, and adhering to prescribed self-management behaviors, among several other pertinent health behaviors. Nonetheless, the novel empirical findings and theoretical advances presented in these studies may be transferable to the maintenance of other health-related behaviors.

## Author contributions

NK drafted the original article. C-QZ and CY revised the draft. All authors contributed to the article and approved the submitted version.
